# Achieving Cure Without Surgery for Olfactory Neuroblastoma: A Case Report and Literature Review

**DOI:** 10.7759/cureus.39614

**Published:** 2023-05-28

**Authors:** Qasim Manzoor Amjad, Mahmood Danishwar, Salman Pervaiz, Satesh Kumar, Giustino Varrassi

**Affiliations:** 1 Internal Medicine, Allama Iqbal Medical College, Lahore, PAK; 2 Gastroenterology and Hepatology, Beth Israel Deaconess Medical Center, Harvard Medical School, Boston, USA; 3 Medicine, Lahore General Hospital, Lahore, PAK; 4 Medicine and Surgery, Shaheed Mohtarma Benazir Bhutto Medical College, Karachi, PAK; 5 Pain Medicine, Paolo Procacci Foundation, Rome, ITA

**Keywords:** surgery, cure, case report, neuroblastoma, olfactory

## Abstract

Olfactory neuroblastoma is a rare, undifferentiated carcinoma of the nasal cavity. It is an extremely rare malignancy, usually occurring in the sixth decade of life with no known underlying cause. In this case report, we present a 71-year-old male with an enlarging facial mass near the right medial nasal bridge, initially diagnosed as undifferentiated carcinoma on biopsy and later confirmed as olfactory neuroblastoma eroding into the anterior skull base. Our patient presented with the signs and symptoms of epiphora, epistaxis, intermittent headaches, anosmia, and an enlarging facial mass. The treatment modalities include surgery, radiation therapy, and chemotherapy. The purpose of this case report is to highlight the importance of chemotherapy and adjuvant radiotherapy for treatment without the need for surgery. Further studies need to be done to divulge the risk factors for olfactory neuroblastoma and to implore new chemotherapeutic treatment modalities that minimize long-term mortality and morbidity.

## Introduction

Olfactory neuroblastoma, also referred to as esthesioneuroblastoma, is a rare malignant tumor of neuroectodermal origin. Berger and Richard first described olfactory neuroblastoma in 1924 [1]. It has been characterized as a rare malignant neuroectodermal neoplasm of the sinonasal cavity, which typically occurs in the superior nasal cavity medial to the middle turbinate and represents about 3% of all sinonasal malignancies [2]. The most accepted site of origin is the olfactory mucosa's basal neural cells, which then arise in the superior portion of the nasal vault [3]. The clinical behavior of olfactory neuroblastoma ranges widely, from relatively indolent to locally aggressive and metastatic. Most of the symptoms are related to the anatomic structures affected by mass effects or local invasion [4].

These lesions can be easily missed because the presenting symptoms mimic those of benign tumors of the nose. The most common symptoms are unilateral nasal obstruction (70%) and epistaxis (50%). Other symptoms include headaches, pain, excessive lacrimation, rhinorrhea, anosmia, and vision changes. Olfactory neuroblastoma originates from the olfactory epithelium; however, olfactory neuroblastoma rarely causes anosmia (5%) [5]. Olfactory neuroblastoma may histologically mimic many types of tumors within the sinonasal tract, making diagnosing it more challenging.

It is challenging for characterization and treatment due to its rare incidence. There are three modalities used to treat olfactory neuroblastoma: surgery, external beam radiation, and chemotherapy. The optimal treatment regimens are still under investigation. Often, a combination of these three modalities is used, namely surgical resection with postoperative irradiation, for all but a small number of tumors [6,7]. Chemotherapy has a role in more advanced cases, but the utility of chemotherapeutic agents is not well defined. For unresectable local diseases, chemoradiation is an approach but lacks strong evidence for the combination of radiation dose and chemotherapy regimen. The present study reports the case of a patient with a mass in the nasal cavity who was treated with combined chemotherapy and radiotherapy because surgery was not possible in his case.

## Case presentation

**Figure 1:**
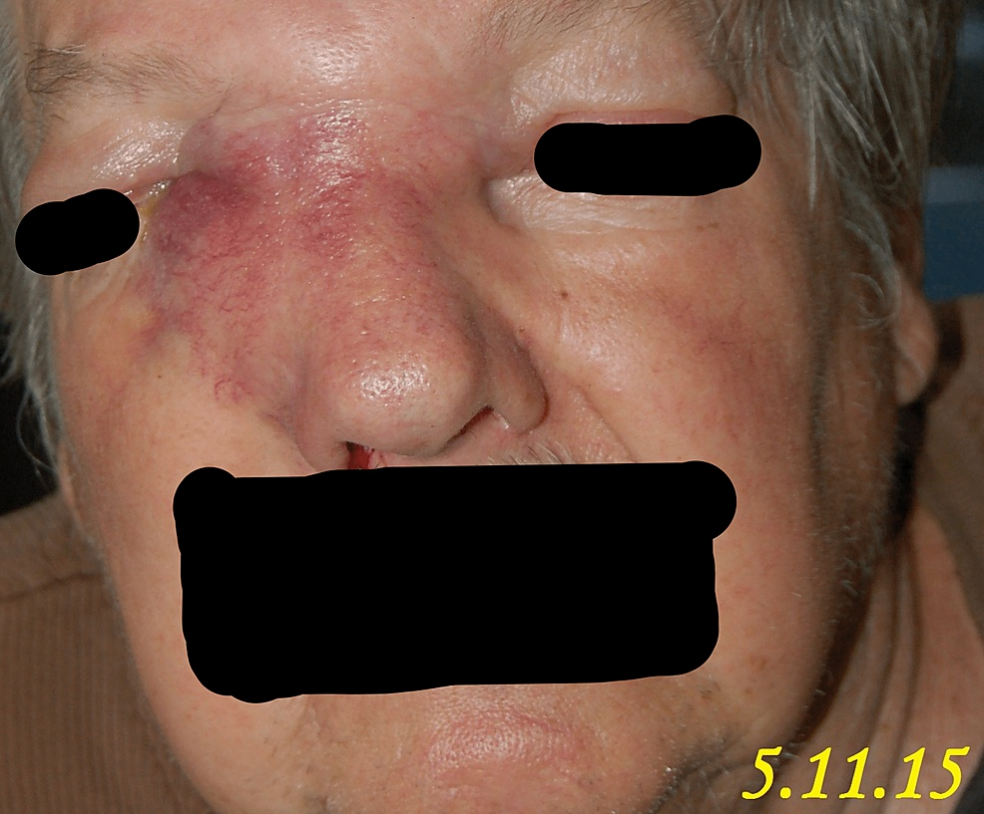
Clinical presentation of a patient with a nasal bridge bump, epiphora, epistaxis, anosmia, and headache: 5.11.15

**Figure 2:**
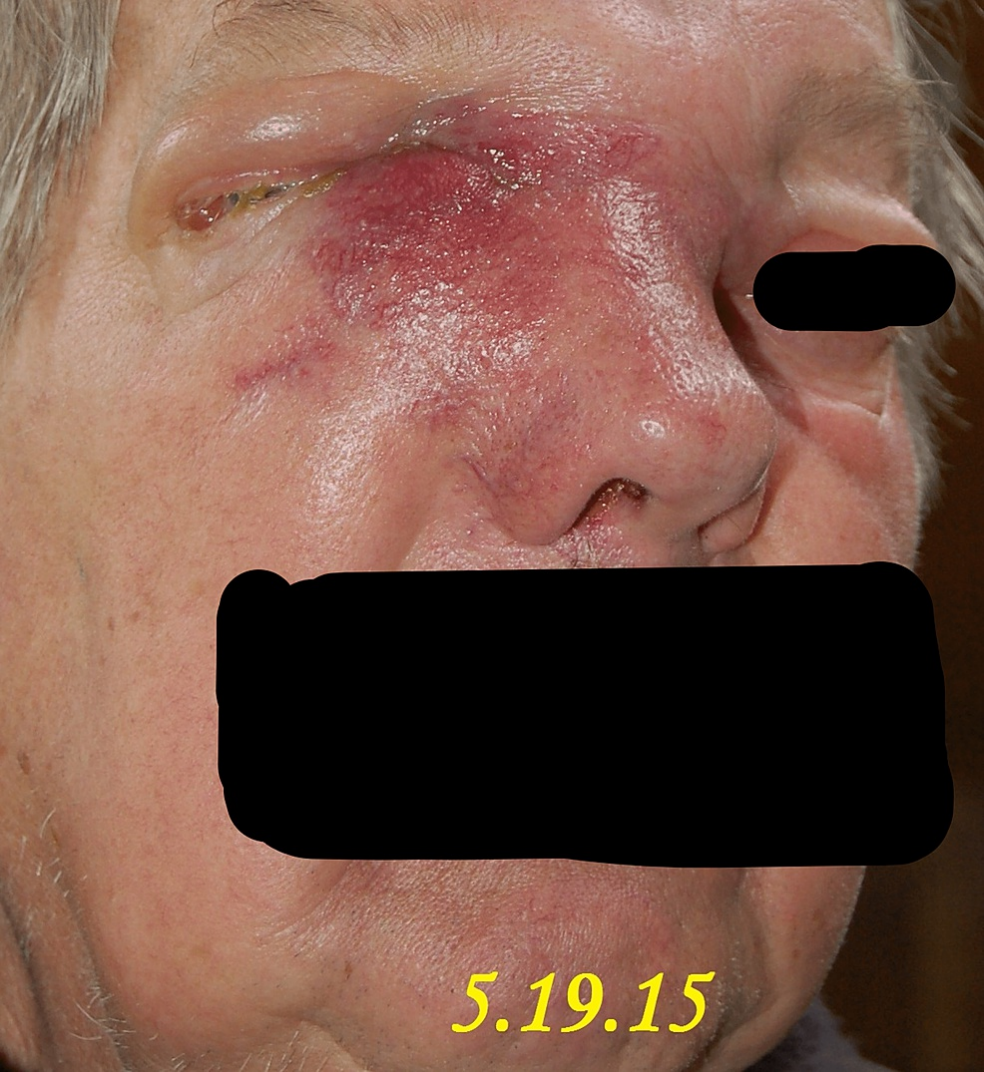
Case study illustrating the clinical features of a patient followed up on 5.19.15, including progressive nasal bridge bump, eye epiphora, epistaxis, anosmia, and headache

**Figure 3:**
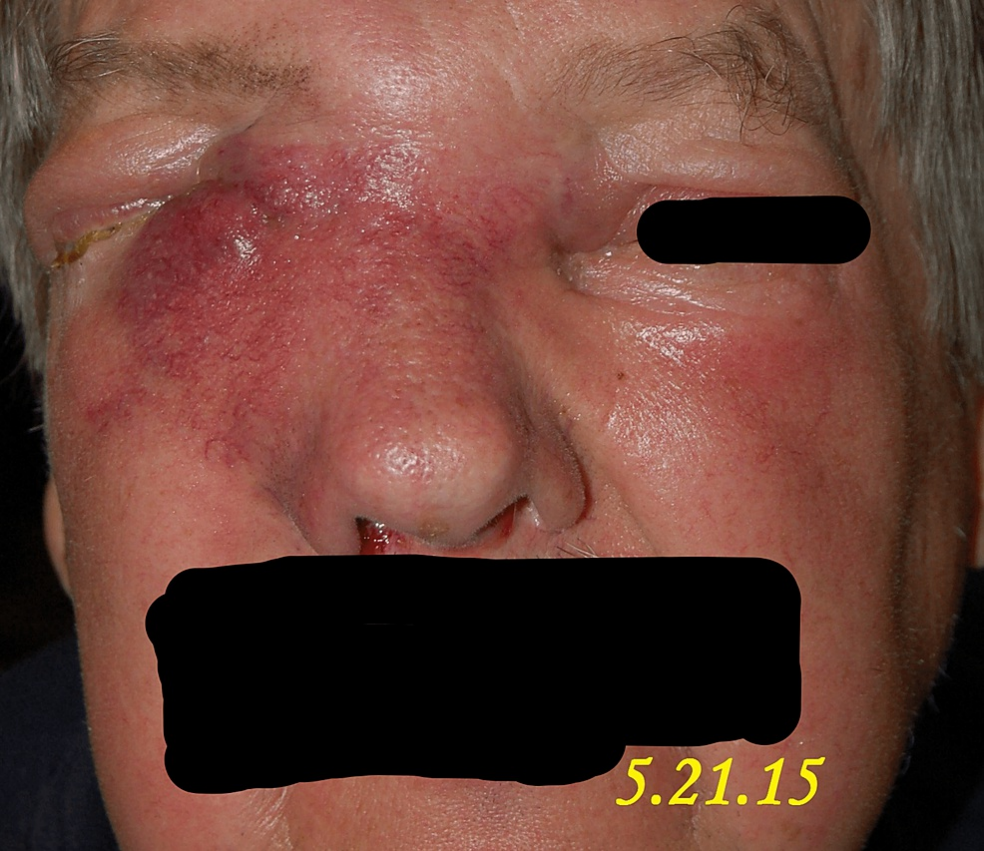
Case study demonstrating the clinical characteristics of a patient monitored on 5.21.15, encompassing advancing nasal dorsum swelling, lacrimation, epistaxis, loss of smell, and cephalalgia

**Figure 4:**
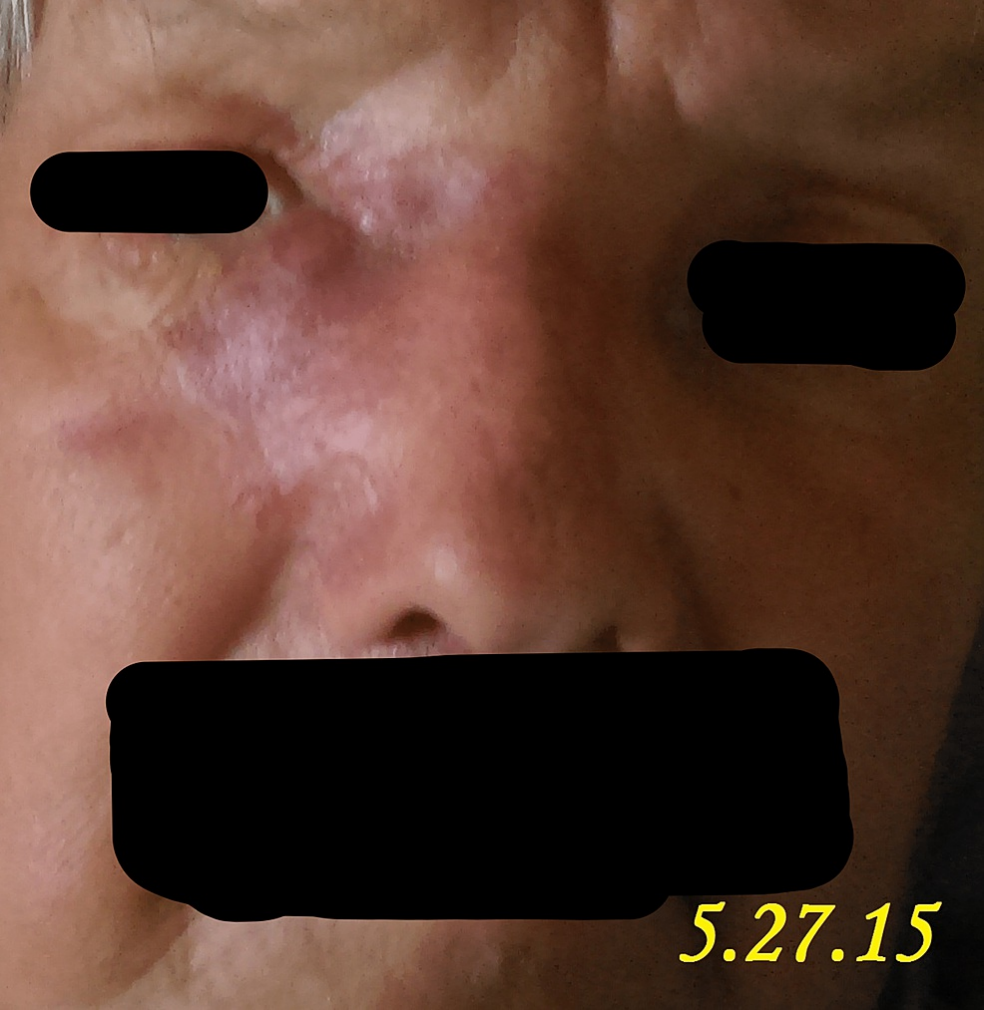
Clinical case study demonstrating the manifestations of a patient monitored on 5.27.15, showcasing advancing nasal bridge swelling, lacrimation, nasal bleeding, loss of smell, and cranial pain

**Figure 5:**
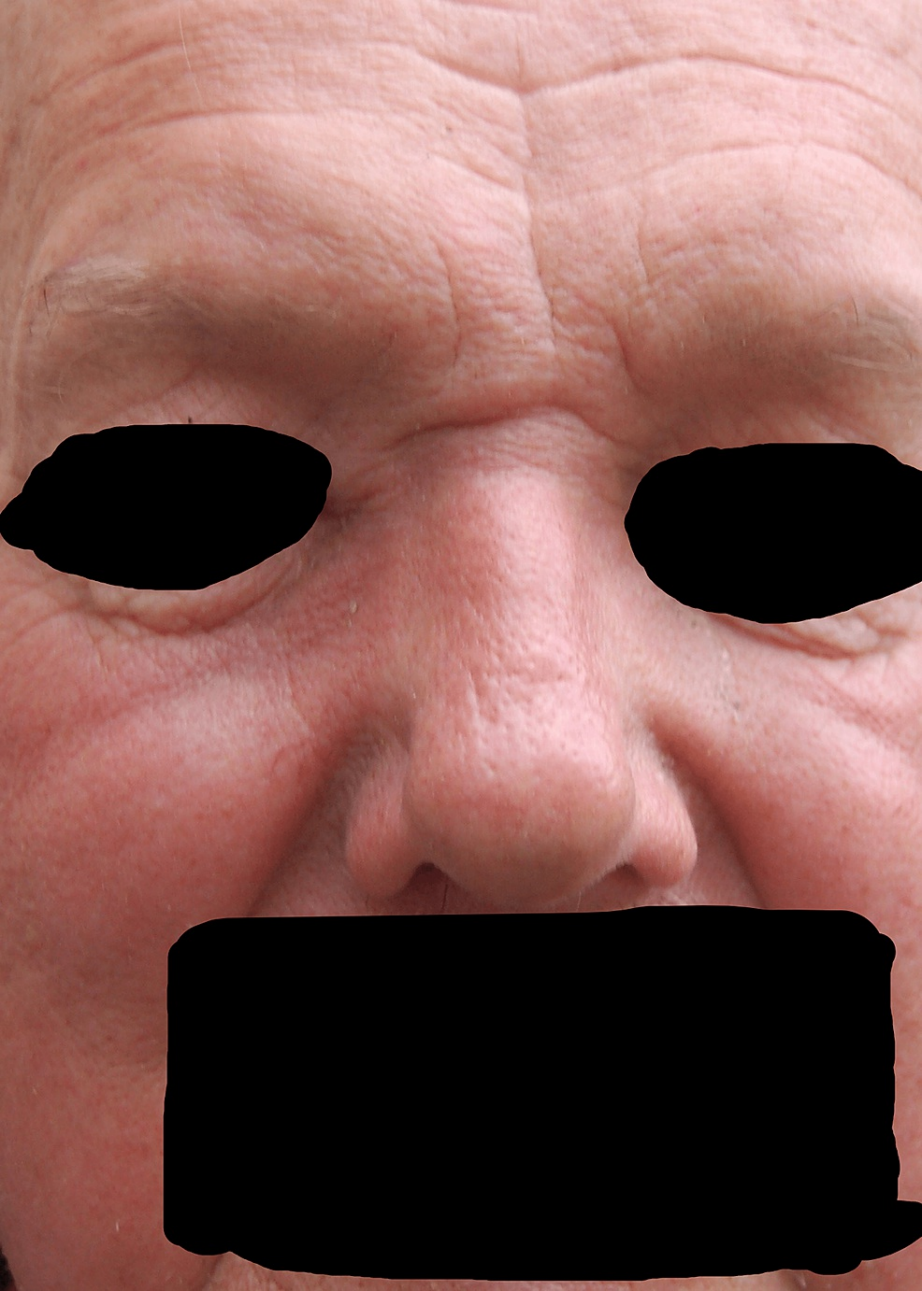
A case study exemplifying the clinical characteristics of a patient observed on 6.10.15, encompassing advancing protrusion on the nasal bridge, excessive tearing from the eyes, nosebleeds, loss of sense of smell, and headaches

A 71-year-old male patient presented to our clinic in April 2015 with the chief complaint of a progressively enlarging bump over the right medial nasal bridge. He had been experiencing right eye epiphora, epistaxis, anosmia, and intermittent headaches for the past month, as shown in Figures 1-5. A biopsy was promptly performed, and the histopathological examination confirmed the diagnosis of olfactory neuroblastoma.

Further imaging investigations, including computed tomography (CT) and magnetic resonance imaging (MRI) scans, revealed a sizeable ethmoidal mass eroded into the medial aspect of the right orbital contents. Notably, it had not invaded the orbital fat, and the integrity of the medial rectus muscle remained unaffected. The imaging also indicated tumor erosion into the anterior skull base, resulting in a mass effect on the brain with a displacement of approximately 15 mm. No spread to the cervical lymph nodes was observed. A positron emission tomography (PET) scan was performed, which demonstrated a right sinonasal mass measuring 5.6 x 5.0 cm with a maximum standardized uptake value (SUV) of 12.8. The tumor infiltrated the right nasal cavity, anterior nasal tissue, right medial orbit, ethmoid air cells, and frontal ethmoidal regions. Considering the extent of the disease and its rapid progression, surgical intervention was deemed unsuitable for the patient. Therefore, a treatment plan involving systemic therapy was initiated. The patient received four cycles of cisplatin plus etoposide, administered over a period of two months (May and June 2015). This was followed by adjuvant radiotherapy. A follow-up MRI conducted after two months of treatment revealed a significant reduction in tumor size, predominantly within the ethmoidal air cells. However, there were concerns regarding the residual disease, as dural enhancement overlaying the frontal lobe and a small area of intracranial tissue at the level of the right ethmoid recess were detected. Subsequent MRI scans performed at six-month intervals over two years demonstrated a progressive decrease in tumor burden. Finally, the MRI conducted in January 2018 showed complete remission of the disease with no evidence of recurrence.

The patient's olfactory neuroblastoma responded well to the treatment regimen, which involved four cycles of cisplatin plus etoposide followed by adjuvant radiotherapy. The imaging findings indicated a significant reduction in tumor size, ultimately leading to complete remission by January 2018, with no signs of disease recurrence. Overall, this case highlights the successful management of olfactory neuroblastoma using a combination of chemotherapy and adjuvant radiotherapy, which proved effective in achieving complete remission without surgical intervention.

## Discussion

Olfactory neuroblastoma (ONB) is a rare malignant neoplasm of the nasal cavity, accounting for approximately 3% of the malignancies of the sinonasal cavity [8]. Although it can occur in any age group, the incidence is highest in the sixth decade of life. There is no gender or race preference for the disease [9]. Localized lesions usually present as unilateral nasal obstruction and epistaxis, whereas lesions extending to the paranasal sinuses or orbital plate can present with diplopia, exophthalmos, lacrimal overflow, and even seizures [10]. Anosmia is a rare symptom occurring in only 5% of patients, and our patient had anosmia upon presentation. Due to non-specific symptoms during the early stages of the disease and the slow progression of the neoplasm, most patients (approximately 70%) present with an advanced stage [11].

In terms of staging, a CT scan helps in determining bone erosions, but an MRI is best for determining the extent and delineation of the tumor. Taking this forward in 1976, Kadish et al. developed a system of staging based on the neuroradiological findings and clinical evaluation, categorizing the tumors into three stages [12]. Stage A is the tumor limited to the nasal cavity, and stage B is the tumor invading one or more paranasal sinuses. Lastly, stage C is when the tumor invades adjacent structures or metastasizes to distant sites. An improvement in the Kadish staging system was suggested by Morita et al. by adding stage D for all diseases with distant metastasis [13]. As per this discussion, the patient presented above is Kadish stage C.

In 1988, Hyam et al. suggested another four-tiered system of staging based on histopathological features and biological factors [14], according to which grades I and II demonstrated low-level tumors and grades III and IV represented high-level tumors. Histologically, ONB consists of small, round blue cells with a nest of neurofibrillary extensions arranged with chromatin dots and having Flexner-Wintersteiner and Homer Wright rosettes [6]. With the increase in the degree of tumor grade, it becomes difficult to make a definite diagnosis, so factors like immunohistochemistry, flow cytometry, molecular studies, and electron microscopy help to confirm the diagnosis. The immunohistochemical markers expressed in ONB are synaptophysin, chromogranin, NSA, CD56, and S-100 [15]. The biopsy report of our patient showed small blue cells with high N:C ratios, hyperchromatism, and an infiltrative pattern. The immunohistochemical staining showed positivity for synaptophysin and chromogranin. Correlating it with clinical findings, a diagnosis of olfactory neuroblastoma was made.

Due to its rarity and anatomic location, there is no set of guidelines in place regarding the ideal treatment course for olfactory neuroblastoma. The most commonly used modalities include surgical resection (craniofacial or endoscopic), radiation therapy, and chemotherapy. For small localized lesions, surgery is enough as a single modality, whereas multiple modalities are used to tackle advanced diseases [7]. Surgical resection combined with postoperative radiotherapy demonstrated the best outcomes in terms of increased survival and a decreased rate of cancer recurrence [16]. The dose of radiation is usually 65-70 Gy in total, given in approximately 35 fractions. Since radiation can cause damage to surrounding structures, the collateral damage can be minimized by the use of intensity-modulated radiation therapy (IMRT). This leads to the sparing of structures such as the brain and optic nerve [17].

In cases of advanced metastatic disease or when there is a medical contraindication to surgery, radiotherapy alone or with adjuvant chemotherapy is used. Usually, platinum-based regimens are used in the treatment of ONB, but the indication of their use and the choice of the best drug are still issues of debate. The most commonly used regimen includes cisplatin plus etoposide given for four cycles [18]. There is a scarcity of data on the subject of the role of chemotherapy as a single modality for the treatment of ONB. Since surgery plus radiotherapy is considered the superior management strategy, there is not enough data on the cure of ONB using the chemo-radiation approach. Jeng et al. reported two such cases in 2019 that were treated with a chemo-radiation approach [19]. One patient was given concurrent radiotherapy and cisplatin chemotherapy, and a decrease in tumor size was reported without recurrence for nine years. The second patient was treated with etoposide plus carboplatin followed by radiotherapy, and he became disease-free without recurrence for eight years. The patient in our case report had a similar course of management, being given cisplatin plus etoposide along with adjuvant radiotherapy, resulting in complete regression and cure of the disease. This case opens up the possibility of a complete cure of advanced ONB with chemo-radiotherapy without the need for surgical intervention.

## Conclusions

As opposed to the prior belief that surgery is the best and first modality for the treatment of olfactory neuroblastomas, this case report of a patient with advanced disease (Kadish stage C) achieving a cure with chemo-radiotherapy alone definitely opens up new horizons in the management of this malignancy. It would be interesting to see the effects of this approach in more patients so that enough data can be gathered and the validity of this treatment can be tested.
